# Quantitative detection of formaldehyde using solid phase microextraction gas chromatography–mass spectrometry coupled to cysteamine scavenging

**DOI:** 10.1038/s41598-023-41609-0

**Published:** 2023-09-05

**Authors:** Sara Y. Chothia, Matthew Carr, Paul S. Monks, Rebecca L. Cordell, Richard J. Hopkinson

**Affiliations:** 1 Leicester Institute for Structural and Chemical Biology and School of Chemistry, University of Leicester, Henry Wellcome Building, Lancaster Road, Leicester, LE1 7RH UK; 2 Space Park Leicester, University of Leicester, 92 Corporation Road, Leicester, LE4 5SP UK

**Keywords:** Cancer, Chemical biology, Chemistry

## Abstract

Formaldehyde (HCHO) is a toxic and carcinogenic pollutant and human metabolite that reacts with biomolecules under physiological conditions. Quantifying HCHO is essential for ongoing biological and biomedical research on HCHO; however, its reactivity, small size and volatility make this challenging. Here, we report a novel HCHO detection/quantification method that couples cysteamine-mediated HCHO scavenging with SPME GC–MS analysis. Our NMR studies confirm cysteamine as an efficient and selective HCHO scavenger that out-competes O-(2,3,4,5,6-pentafluorobenzyl)hydroxylamine, the most commonly used scavenger, and forms a stable thiazolidine amenable to GC–MS quantification. Validation of our GC–MS method using FDA and EMA guidelines revealed detection and quantification limits in the nanomolar and micromolar ranges respectively, while analysis of bacterial cell lysate confirmed its applicability in biological samples. Overall, our studies confirm that cysteamine scavenging coupled to SPME GC–MS analysis provides a sensitive and chemically robust method to quantify HCHO in biological samples.

## Introduction

Formaldehyde (HCHO) is a highly reactive environmental pollutant that is toxic and carcinogenic to humans at high concentrations or after prolonged exposure^1^. Environmental HCHO exposure can arise from multiple sources including from plastics manufacturing^2^, wood products^3^, textiles^4^ and paints^2^. Additional sources include food, particularly processed food, soya beans and fruits^5,6^, and cosmetics, which often contain HCHO-releasing additives^7^.

In addition to environmental exposure, human cells are exposed to HCHO as a product of metabolism. Important pathways producing this endogenous HCHO are tetrahydrofolate degradation^8,9^ and enzymatic demethylation reactions, which regulate methylation levels on proteins and nucleic acids in humans and other organisms^10–12^. Many if not all of the metabolic processes producing HCHO are constitutively active, which suggests that cells must be able to tolerate HCHO exposure. Recent evidence additionally suggests that HCHO, when present at sub-toxic levels, has healthy biological functions, for example as a carbon source in folate metabolism^9^. Therefore, balancing HCHO’s healthy and toxic effects, e.g. by regulating intracellular HCHO concentrations, is essential for maintaining human health.

From a chemical perspective, HCHO is a highly reactive electrophile that forms multiple structurally distinct adducts with nucleophilic biomolecules such as proteins and nucleic acids^13–15^. These adducts, which include hydroxymethylated and cross-linked species, have varying formation rates and stabilities. In most cases, HCHO-derived biomolecule adducts are presumed to be responsible for HCHO’s biological functions; however, (bio)chemical evidence is often lacking.

Defining the complex biology of HCHO requires methods that can detect and quantify it in biological samples. To do this effectively, methods must be able to either (i) identify and quantify free HCHO and all HCHO-derived adducts in the sample, or (ii) use HCHO scavenging molecules to out-compete other species for reaction with HCHO, and then quantify the resultant scavenger-HCHO adducts. Given the efficient and promiscuous reactivity of HCHO, both approaches are very challenging; however, some success has been reported with the second approach using scavengers containing predominantly amine (including 1,2-diamine), hydroxylamine, hydrazine or 4-amino-alkene groups^16–36^. While these groups often give some selectivity for HCHO over other biologically relevant reactive carbonyls (variable) and have been used to quantify HCHO levels in cells and tissue, they are often slow to react with HCHO and only form quasi-stable adducts. This means they are unable to completely sequester all HCHO from a sample.

**Figure 1:**
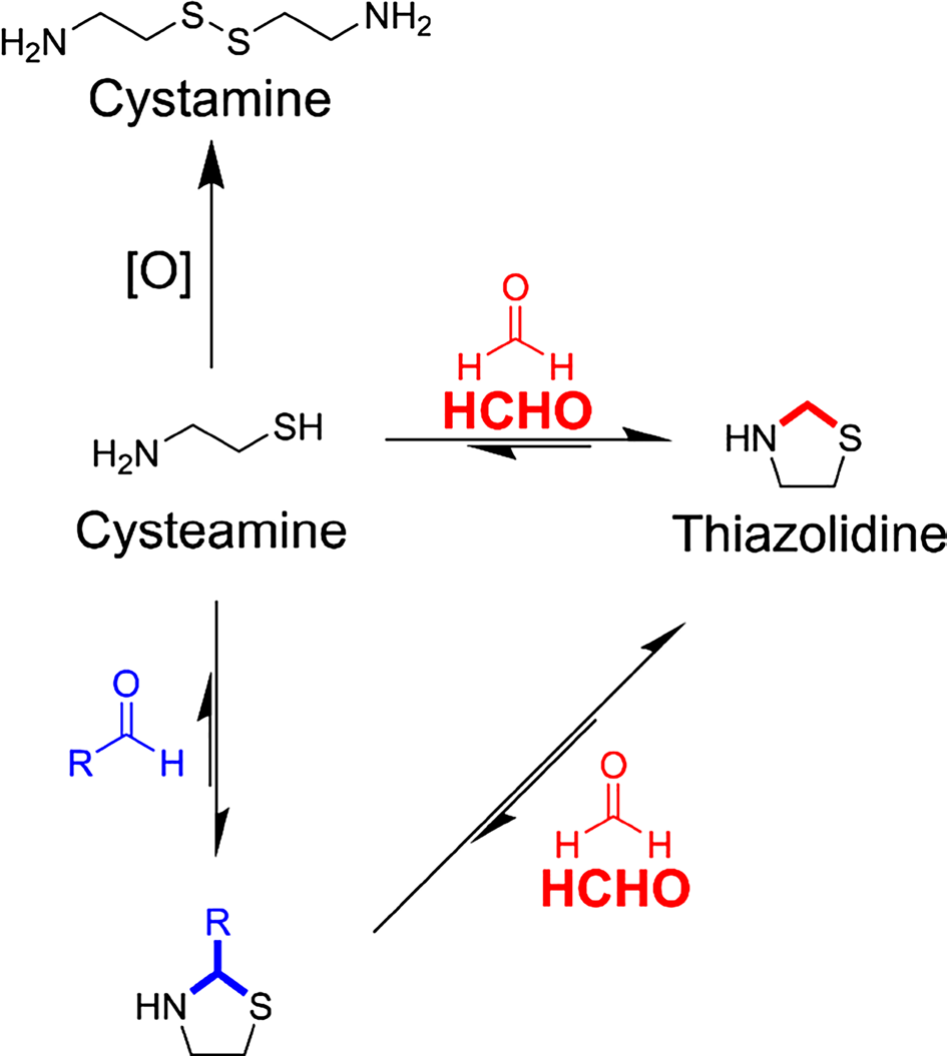
Cysteamine scavenges formaldehyde (HCHO) and other aliphatic aldehydes to form thiazolidines under physiologically relevant conditions. HCHO forms the most stable thiazolidine and can out-compete other aldehydes for reaction with cysteamine. Cysteamine can also undergo irreversible oxidation to cystamine.

Here, we report the development of a HCHO quantification method that uses cysteamine as a HCHO scavenger coupled to solid phase microextraction (SPME) and gas chromatography-mass spectrometry (GC–MS) analysis. We selected GC–MS as an attractive method for analysing HCHO as it is highly sensitive and uses MS-based detection, which should give a robust identification of the HCHO-derived adduct that can be further validated using an authentic standard. GC–MS has been widely used to detect volatile molecules from aqueous samples^37–39^, and while not generally suitable for detecting HCHO directly, has been used to detect and quantify volatile HCHO-derived adducts with scavengers, including with cysteamine using direct injection^18,35,36^. However, this method required large sample volumes and a lengthy preparation protocol that involved liquid–liquid extraction of the HCHO-derived adduct. Conversely, coupling GC–MS analysis to SPME, where the HCHO-derived adduct is adsorbed onto the SPME fibre and then desorbed prior to MS analysis, should result in shorter and simpler sample preparation procedures. Such a method might also provide improved sensitivity over direct headspace injection methods, which have been reported for HCHO detection using the HCHO scavenger O-(2,3,4,5,6-pentafluorobenzyl)hydroxylamine (PFBHA)^19,20,22–24,33^. The improved sensitivity of SPME methods should additionally enable analysis of smaller sample sizes. Our selection of cysteamine as a HCHO scavenger was informed by its use in previous GC–MS studies and also by recent work on the structurally related amino acid cysteine, which was shown to be an efficient and selective scavenger for HCHO in NMR- and LC–MS-based analyses (Fig. 1)^1, 40^.

Overall, these studies resulted in the development of a novel HCHO analysis method capable of HCHO detection in the nanomolar range and quantification in the micromolar range.

## Results

Initial experiments focused on determining the suitability of cysteamine as a HCHO scavenger. For cysteamine to be considered suitable for HCHO scavenging and GC–MS applications, its reaction with HCHO must be fast and selective, while its reaction product(s) must be sufficiently stable to survive heating (as occurs during GC–MS sample extraction) and to disfavour formation of HCHO-derived adducts with competing nucleophiles. For headspace GC–MS methods, where the analyte is driven from the liquid sample into the headspace by heating, the cysteamine-HCHO adduct must also be suitably volatile. This is not the case with cysteine.

**Figure 2:**
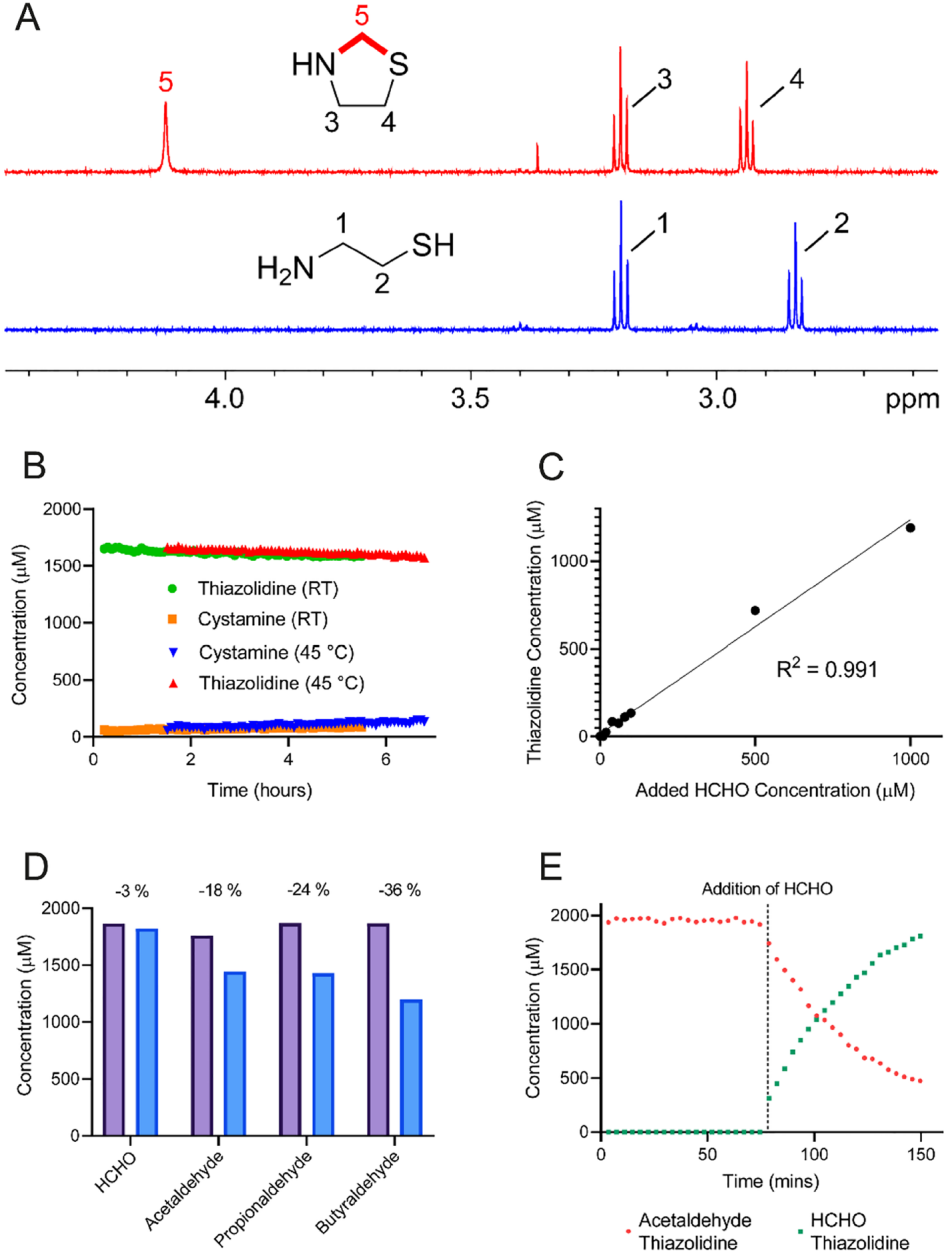
^1^H NMR analyses on the reactions of cysteamine with aldehydes. (**A**) ^1^H NMR spectra of cysteamine (blue) and HCHO-derived thiazolidine (red). The thiazolidine was formed by incubation of cysteamine with a tenfold excess of HCHO. (**B**) Graph showing time-dependent degradation of HCHO-derived thiazolidine and concomitant formation of cystamine over time. Heating of the thiazolidine at 45 °C for one hour (as occurs during immersive GC–MS analysis) did not affect stability relative to incubation at room temperature (RT). (**C**) Graph showing calculated HCHO-derived thiazolidine concentrations from samples of cysteamine and different concentrations of HCHO. A linear correlation between added HCHO concentration and thiazolidine concentration was observed (R^2^ = 0.991). (**D**) Bar graph showing thiazolidine concentrations in samples of cysteamine and different aldehydes (10-fold excess). Near-full conversion of cysteamine to the respective thiazolidines was observed after one hour (purple); however, the HCHO-derived thiazolidine was the most stable over 24 h (blue). (**E**) Graph showing thiazolidine concentrations in a sample of cysteamine first incubated with acetaldehyde (10-fold excess) followed by addition of HCHO (100-fold excess). In the absence of HCHO, full conversion to the acetaldehyde-derived thiazolidine is observed; however, addition of HCHO leads to rapid conversion to the HCHO-derived thiazolidine.

An initial NMR time-course experiment was conducted on a sample containing cysteamine (2 mM) and 10 equivalents of HCHO in 100 mM sodium phosphate buffer pH 7.4 (containing 25% v/v D_2_O) at 25 °C. Reaction between cysteamine and HCHO was completed before the first NMR timepoint (301 s after mixing) to form a product with ^1^H NMR resonances at δ_H_ 2.94 ppm (triplet), δ_H_ 3.20 ppm (triplet) and δ_H_ 4.12 ppm (singlet) respectively (Fig. 2A). The product was assigned as cysteamine thiazolidine on the basis of 2-dimensional NMR analyses and by comparison with an authentic standard (Fig. S1). While it is possible that additional hydroxymethylation occurs on the nitrogen or sulphur of cysteamine or on the nitrogen of the thiazolidine (note: HCHO was present at a 10-fold excess), no evidence for these species was accrued in the NMR spectra. However, it is possible that hydroxymethylation is too dynamic for NMR detection or that its corresponding NMR resonances are obscured by overlap or water suppression. A control experiment omitting HCHO revealed formation of a different product derived from cysteamine with two characteristic triplet ^1^H NMR resonances at δ_H_ 3.04 ppm and δ_H_ 3.40 ppm respectively (Fig. S2). This species was assigned as cysteamine disulfide (cystamine), which is formed by cysteamine oxidation. However, cystamine formation was only 50% completed after one hour, which suggests that oxidation is a much slower process than thiazolidine formation when HCHO is present in excess.

The stability of the thiazolidine product was then assessed. In the sample with 10 equivalents of HCHO, partial degradation of the thiazolidine was observed over the 5-h time-course, which correlated with low-level formation of cystamine (Fig. S3A). To determine whether excess HCHO or cysteamine concentrations affect thiazolidine stability, a new sample was prepared containing authentic thiazolidine (2 mM), which revealed faster rates of thiazolidine degradation and cystamine formation (Fig. 2B). However, thiazolidine degradation was still slow under the tested conditions (12.7 µM hour^−1^, Fig. 2B) and was comparable to the degradation rate observed with excess HCHO (13.6 µM hour^−1^, Fig. S3A). Repeating the initial experiment but with 0.5 equivalents of HCHO relative to the initial cysteamine concentration (2 mM) revealed faster cystamine formation but a slower rate of thiazolidine degradation (6.1 µM hour^−1^), which might indicate a concentration dependence on thiazolidine degradation (Fig. S3B). Collectively, these studies suggest that HCHO-derived thiazolidine is a quasi-stable product, forming an equilibrium with free HCHO and cysteamine. The cysteamine can then undergo slow irreversible oxidation, thus draining the equilibrium towards the cystamine product (Fig. 1). However, the slow rates of thiazolidine degradation and cystamine formation suggest that cysteamine is a suitable HCHO scavenger for quantification experiments over short analysis periods (hours).

We then tested the stability of the thiazolidine after sample heating. Samples were prepared containing cysteamine (1.67 mM) and a 10-fold excess of HCHO in 100 mM sodium phosphate buffer pH 7.4 (containing 25% v/v D_2_O) at 25 °C, and were then heated at either 80 °C or 100 °C for 20, 30, 40 or 70 min before NMR analysis. Thiazolidine degradation (and concomitant cystamine formation) was more significant at higher temperatures and after longer heating times (Fig. S4). However, the most significant thiazolidine degradation, which was observed after heating at 100 °C for 70 min, was still small relative to the initial thiazolidine concentration (27% degraded at 24 h after heating). Similar low-level degradation was observed in samples with 0.5 equivalents of HCHO (Fig. S5). Heating of a sample of authentic thiazolidine at 45 °C for 70 min also did not reveal significant degradation relative to treatment at room temperature (thiazolidine degradation rate = 13.5 µM hour^−1^, Fig. 2B). Therefore, heating of thiazolidine-containing samples, at least for short periods, should enable sensitive detection of HCHO by GC–MS.

We then assessed the efficiency of thiazolidine formation at varying concentrations of HCHO. For these experiments, cysteamine (2 mM) was mixed with varying concentrations of HCHO (1 µM–1 mM) in 100 mM sodium phosphate buffer pH 7.4 (containing 25% v/v D_2_O) and the reactions were monitored by ^1^H NMR at 25 °C. At HCHO concentrations below 10 µM, no product was observed, which is likely due to insufficient sensitivity of the NMR analysis method. However, in the other samples, thiazolidine formation was completed at the first NMR timepoint, while analysis of the HCHO concentrations at this timepoint revealed a linear correlation between initial HCHO concentrations and the concentrations of thiazolidine product (coefficient of determination, R^2^ = 0.991, Fig. 2C).

We then determined the selectivity of cysteamine for HCHO over other carbonyl compounds. Initially, the reaction of cysteamine with five representative carbonyl compounds (acetaldehyde, propionaldehyde, butyraldehyde, glyoxylic acid and acetone) were analysed by ^1^H NMR under conditions analogous to those used with HCHO (10 equivalents of carbonyl relative to cysteamine). No reaction was observed with acetone; however, time-course experiments revealed reactions between cysteamine and the other carbonyls at rates comparable to that observed with HCHO (Fig. S6). These studies suggest that cysteamine is an unselective carbonyl scavenger, which implies that its use for HCHO scavenging might be compromised by the presence of other carbonyls in biological (or other) samples. However, analysis of the samples after 24 h revealed, at least for acetaldehyde, propionaldehyde and butyraldehyde, that the reaction products (presumed to be thiazolidines by comparison with HCHO studies) were less stable than the HCHO-derived thiazolidine (Fig. 2D). This observation was supported by competition experiments, where cysteamine (2 mM) was pre-incubated with the carbonyl compound (10 equivalents) before addition of 100 equivalents of HCHO. With the aliphatic aldehydes acetaldehyde, propionaldehyde and butyraldehyde, full conversion to the cysteamine-aldehyde product was observed in the pre-incubation period; however, rapid conversion of these species to the HCHO-derived thiazolidine was observed after addition of HCHO (Figs. 2E and S7). Reciprocal experiments, where cysteamine was pre-incubated with 10 equivalents of HCHO before addition of aliphatic aldehyde (100 equivalents), did not reveal formation of cysteamine-aldehyde products. These results suggest that the HCHO-derived thiazolidine is stable under the tested conditions. Collectively, the experiments with other carbonyls imply that cysteamine, while reacting efficiently with aliphatic aldehydes in isolation, is selective for HCHO in mixtures owing to the reduced dynamicity of the cysteamine/HCHO—thiazolidine equilibrium. This is important because it suggests that cysteamine will not be sequestered by other carbonyls present in complex mixtures, thus ensuring efficient scavenging of HCHO. Selectivity for HCHO should also preclude significant formation of structurally similar adducts with other carbonyls that might affect adsorption of the HCHO-derived thiazolidine onto the SPME fibre and signal resolution during GC–MS analyses. However, with glyoxylic acid, no interconversion was observed in the competition experiments, which suggests that both glyoxylic acid and HCHO can be scavenged by cysteamine with similar efficiencies. Quantitative analyses must therefore be able to distinguish between the HCHO-derived and glyoxylic acid-derived adducts, which is achievable using GC–MS given their different masses.

We then compared the relative reactivity of cysteamine and PFBHA, which is a widely used aldehyde scavenger in GC–MS-based detection experiments^17,33^. We conducted ^1^H NMR time-course studies monitoring the reaction of PFBHA (2 mM) with HCHO (20 mM or 1 mM) under analogous conditions to those used for cysteamine. Formation of the PFBHA-HCHO adduct (an oxime) was evidenced by the emergence of new ^1^H resonances at δ_H_ 5.3 ppm, δ_H_ 6.6 ppm and δ_H_ 7.1 ppm; however, formation of this adduct was markedly slower than the formation of the cysteamine-HCHO thiazolidine adduct under equivalent conditions (Fig. S8). Stability studies after heating at 45 °C, 80 °C and 100 °C revealed temperature-dependent loss of the oxime adduct; while this might be due to degradation back to PFBHA, it is also possible that the adduct is evaporating from the reaction mixture, thus reducing the detectable concentration (note: PFBHA and HCHO cannot be detected by ^1^H NMR under these conditions due to signal overlap, Fig. S8). Two competition experiments were also conducted that involved a pre-incubation of one scavenger (cysteamine or PFBHA, 5 equivalents) with HCHO, before addition of 50 equivalents of the other scavenger. When cysteamine was added first followed by PFBHA, cysteamine-derived thiazolidine formation was observed and remained at constant concentration after PFBHA addition, while no discernible evidence of the PFBHA-HCHO adduct was apparent (Fig. S8D). However, both the PFBHA- and cysteamine-derived adducts were observed when PFBHA was added first, followed by cysteamine (Fig. S8D). The PFBHA-derived oxime decreased in concentration after addition of cysteamine (35% reduction after 2 h). These experiments therefore imply that cysteamine can out-compete PFBHA for reaction with HCHO under the tested conditions.

**Figure 3:**
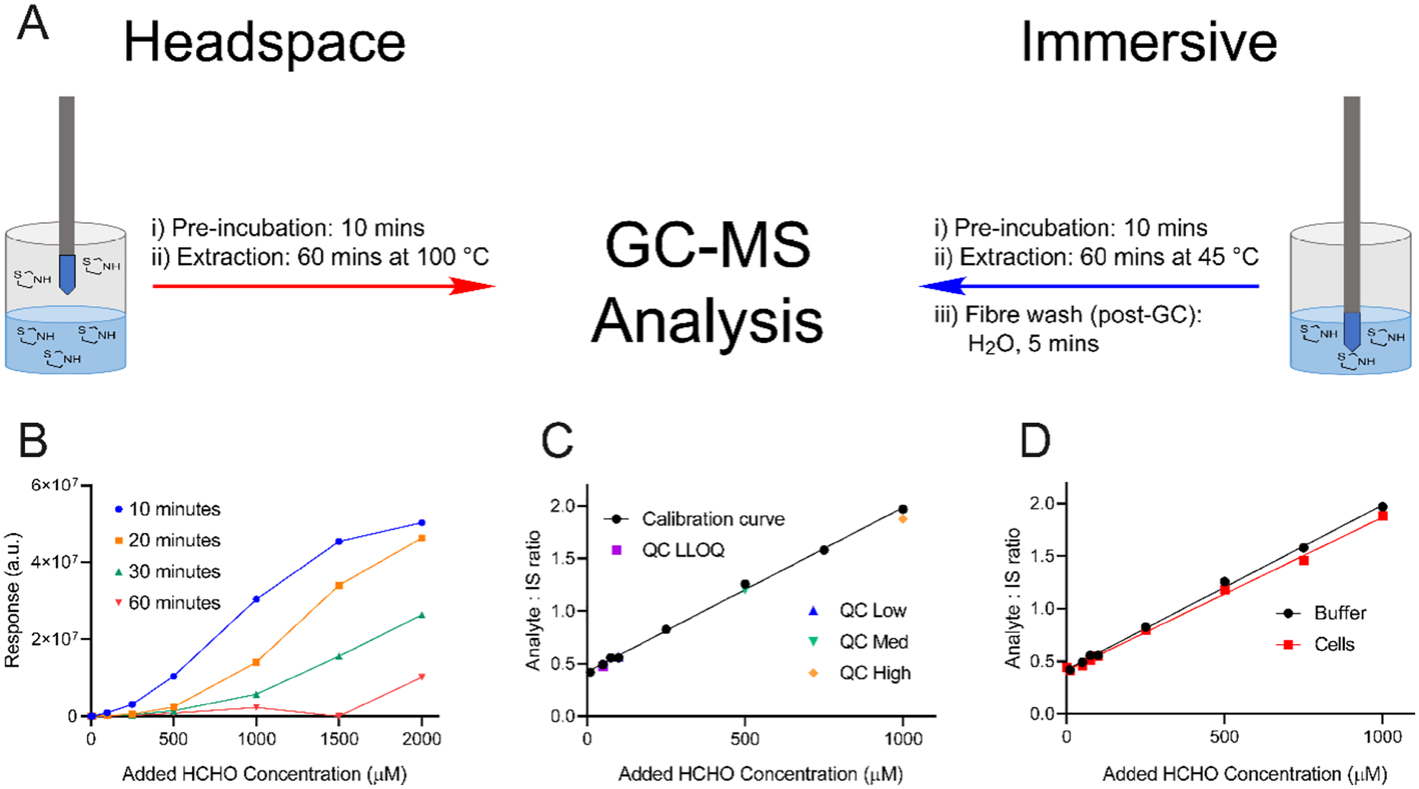
Detection of HCHO using cysteamine scavenging coupled to SPME GC–MS analysis. (**A**) Scheme showing protocols for headspace (left) and immersive (right) extraction and GC–MS analysis of HCHO-derived thiazolidines. In both cases, the sample is initially heated to equilibrate its temperature (pre-incubation, 10 min). The SPME fibre is then added to enable extraction of the HCHO-derived thiazolidine. After the extraction period, the fibre is then removed from the sample vial and heated to desorb the thiazolidine for GC–MS analysis. (**B**) Graph showing headspace GC–MS response curves from samples of cysteamine incubated with HCHO. The samples were heated at 100 °C for either 10, 20, 30 or 60 min to induce evaporation of thiazolidine into the headspace and adsorption onto the SPME fibre (note: the samples were also subjected to a pre-incubation step involving heating at 100 °C for 10 min). a.u = arbitrary units. (**C**) Calibration curve of HCHO-derived thiazolidine in 100 mM sodium phosphate buffer pH 7.4 (R^2^ = 0.998). Quality control (QC) values are highlighted. Note error bars are too small to be observed. (**D**) Calibration curve of HCHO-derived thiazolidine in *E. coli* cell lysate. The curve (red) was near-identical to that observed with buffer (black). Note error bars are too small to be observed.

Efforts then focused on using cysteamine as a scavenger in SPME GC–MS-based HCHO quantification experiments. Initially, method development employed headspace SPME GC–MS, which relies on the HCHO-derived thiazolidine being evaporated from the aqueous sample (requiring heating and agitation) before adsorption onto an SPME fibre suspended in the headspace. The fibre is then inserted into the heated inlet of the gas chromatograph to induce desorption of the thiazolidine and to enable subsequent chromatographic separation and MS analysis (Fig. 3A). Optimal sensitivity for HCHO detection in 100 mM sodium phosphate buffer pH 7.4 was achieved using sample volumes of 500 µL subjected to heating at 100 °C for 10 min and agitation at 500 rpm (Figs. 3B and S9). Importantly, analysis of the raw spectra revealed detectable signals for the thiazolidine at all HCHO concentrations tested, with even the least concentrated samples (1 nM) giving responses with signal-to-noise ratios ≥ 3 (lower limit of detection, LLOD). However, analysis at different HCHO concentrations revealed a sigmoidal response across the concentration range. Efforts therefore moved to immersive extraction, where the SPME fibre is inserted into the sample to enable thiazolidine adsorption (Fig. 3A). This method does not require evaporation of the thiazolidine from the solution, thus minimising thiazolidine degradation and potentially improving adsorption efficiency. An initial calibration curve using this extraction technique gave a linear response between HCHO concentrations of 10 µM and 1 mM (R^2^ = 0.987). Signals corresponding to the thiazolidine were observed in all samples down to 1 nM (the lowest concentration tested); however, no discernible change in response was observed in samples below 10 µM (Fig. S10).

The precision, accuracy, linearity and sensitivity of the immersive method were then investigated as part of a validation procedure following Food & Drug Administration and European Medicines Agency guidelines^41,42^. For these experiments, the sample preparation protocol remained identical to the initial immersive GC–MS experiments; however, ^13^C-labelled thiazolidine (produced by incubating cysteamine with ^13^C-labelled HCHO) was added to the samples after addition of cysteamine, which enabled more accurate quantification by providing an internal standard with a different mass. In brief, analysis was conducted on a series of 8 calibration standards containing HCHO in 100 mM sodium phosphate buffer pH 7.4 (10 µM to 1 mM), as well as on quality control (QC) samples that were prepared at four concentrations (QC Low (50 µM), QC Medium Low (100 µM), QC Medium High (500 µM) and QC High (1 mM)). The QC samples, which consisted of 6 replicates for each concentration, were prepared using different HCHO stocks to those used to prepare the calibration standards (for full details, see the Supplementary Information). To determine the linearity of the method, the calibration standards were analysed in duplicate and the R^2^ value was determined for an average of the two calibration curves. This value (0.998) was higher than the pre-defined acceptance criterion R^2^ value of 0.950 (Fig. 3C and Table 1). QC samples were used to assess the intra-batch precision (co-efficient of variation, or %CV) and accuracy (relative error, or %RE) of the method. For the batch to pass acceptance criteria, three of the four QC levels were required to be ± 15% of nominal concentrations and ± 20% at QC Low for both %CV and %RE (equations used to calculate %CV and %RE are given in the Supplementary Information). Calculated %CV and %RE values for the immersive method over the validated concentration range (between QC Low and QC High, 50 µM–1 mM) passed acceptance criteria, with only one QC level (QC Low) giving a %RE value greater than 20% (Table 2). Calculation of %CV and %RE values for the calibration samples at 10 µM were ± 20%, which suggests that this concentration is not linearly quantifiable (Table 1). Interestingly, signals for the thiazolidine were observed in control samples without added HCHO, which implies that the buffer contains detectable levels of residual HCHO. Residual HCHO was detected in different control samples using two separately prepared buffer stocks, which suggests that HCHO is a common contaminant in aqueous solutions. While the concentration of this residual HCHO is below our validated concentration range (< 50 µM), its presence suggests that actual detectable HCHO concentrations (for these experiments and potentially in other studies) are likely to be higher than the concentrations of HCHO added to the samples.

**Table 1:** Back-calculated concentrations of HCHO in calibration standards analysed as part of a validation assessment of the immersive SPME GC–MS HCHO detection method.

Conc. (µM)	Conc 1. (µM)	Conc 1. (µM)	Mean Conc. (µM)	S.D	%CV	%RE
10	− 7.15	2.00	− 2.58	4.58	− 177	− 126
50	53.2	34.0	43.6	9.58	22.0	− 12.8
75	93.8	73.8	83.8	10.0	11.9	11.8
100	83.3	83.3	83.3	0	0	− 16.7
250	254	250	252	2.23	0.883	0.869
500	530	513	521	8.75	1.68	4.25
750	730	716	723	6.60	0.913	− 3.61
1000	969	962	966	3.60	0.373	− 3.45

*Conc.* added HCHO concentration, *Conc. 1/2* HCHO concentrations for replicates 1 and 2 back-calculated from the calibration curve, *Mean Conc.* mean of Conc. 1 and Conc. 2, *S.D.* standard deviation, *%CV* % co-efficient of variation, *%RE* % relative error, Number of replicates = 2.

**Table 2:** Back-calculated concentrations of HCHO in QC samples analysed as part of a validation assessment of the immersive SPME GC–MS HCHO detection method.

Conc. (µM)	Mean Conc. (µM)	S.D	%CV	%RE
QC low 50	34.8	6.76	19.4	− 30.4*
QC medium low 100	91.2	6.74	7.39	− 8.79
QC medium high 500	487	4.04	0.829	− 2.63
QC high 1000	908	11.4	1.25	− 9.18

*Conc.* added HCHO concentration, *n* number of replicates, *Mean Conc.* Mean HCHO concentration for replicates 1–6 back-calculated from the calibration curve, *S.D.* standard deviation, *%CV* % co-efficient of variation, *%RE* % relative error, Number of replicates = 6.

* > 20%.

Finally, we tested the validated method with lysate from *Escherichia coli* BL21(DE3) cells. For these experiments, varying amounts of HCHO (10 µM–1 mM, each in triplicate) were added to aliquots of lysate (in 100 mM sodium phosphate buffer pH 7.4) that had been previously subjected to DNA digestion (using DNase 1) and protein precipitation by addition of acetonitrile. Cysteamine was then added followed by addition of ^13^C-labelled thiazolidine as the internal standard. GC–MS analysis revealed a linear response that was near-identical to that observed with buffer only (Fig. 3D). Identical trends were also observed when analysing samples diluted prior to thiazolidine extraction (Fig. S11). No evidence for lysate-derived HCHO was accrued in any of the samples, which might indicate low HCHO levels in these cells and/or that HCHO was removed during sample preparation (e.g. during the protein precipitation step). However, the similar responses observed in lysate and buffer-only samples suggest that cysteamine can sequester HCHO in the presence of competing biological nucleophiles, and that adsorption/desorption of thiazolidine onto/from the SPME fibre is not compromised in the lysate. Therefore, the method appears amenable to the analysis of biological samples.

## Discussion

Quantification of HCHO from biological samples is essential for determining its biological functions but this is hindered by a lack of robust scavenging and analysis methods. Our validation and application of cysteamine as a HCHO scavenger in immersive SPME GC–MS provides a novel and chemically robust technique amenable to detection of HCHO in the nanomolar range and quantification in the micromolar range, including in bacterial cell lysate. The quantification limit (50 µM) calculated for our method is comparable to those reported for most HCHO detection methods, including for the previously reported GC–MS methods using cysteamine^27,34,36,43^. While some studies report lower quantification limits than those possible with our method, these approaches use either larger sample volumes and/or employ PFBHA, which was a less efficient HCHO scavenger than cysteamine in our competition experiments (Fig. S6)^16–26,28–30,33,35^. Direct comparison between methods is also complicated by the use of different statistical analysis protocols, which can result in different definitions for quantification. Importantly, reported limits for HCHO detection (LLOD) might also be complicated by residual HCHO arising from environmental contamination or degradation of sample components.

Overall, our studies confirm that coupling cysteamine scavenging to GC–MS analysis offers a sensitive and robust approach for HCHO detection and quantification that is amenable to biological studies. Given the wide availability of GC–MS instruments and its reproducible ionisation, the method should therefore be generally applicable to HCHO detection in many contexts. Also, the efficiency and relative selectivity of cysteamine for HCHO observed in our NMR studies highlights the as-yet unrealised potential of cysteamine and related 1,2-aminothiols in HCHO detection methods employing GC–MS and other analytical techniques. There is also potential for HCHO scavenging by cysteamine to be of biomedical relevance, for example, in treatments for HCHO exposure or for diseases with elevated endogenous HCHO concentrations.

## Methods

### Reagents

All buffers and media used for NMR and GC–MS analysis were prepared in-house using typical procedures. HCHO stocks were prepared by cracking paraformaldehyde (Fisher) in milliQ water by heating at 60 °C until the solution became clear. Internal standard solutions were prepared by reacting 20 mM cysteamine (Alfa Aesar) with 10 mM ^13^C-labelled HCHO (Santa Cruz Biotechnology) in milli-Q water to form the ^13^C-labelled thiazolidine in situ. All other reagents were purchased from commercial suppliers as follows: sodium phosphate monobasic monohydrate (Insight Biotechnology), sodium phosphate dibasic anhydrous (MP Biomedicals), D_2_O (Cambridge Isotope Laboratories), DMSO-d_6_ (Cambridge Isotope Laboratories), 3(trimethylsilyl)propionic-2,2,3,3-d_4_ acid sodium salt (TSP, Alfa Aesar), thiazolidine (Fluorochem), PFBHA (Sigma Aldrich), acetonitrile (Fisher), LB broth (Melford), LB/Agar (Merck), BL21 (DE3) cells (Agilent), protease inhibitor (EDTA-free) tablets (Fisher Scientific), DNase 1 (Merck) and lysozyme (SLS).

### NMR experiments

#### General

All cysteamine/thiazolidine NMR experiments were conducted on a Bruker 500 MHz spectrometer equipped with a 5 mm BBO probe and TopSpin 3.5 software. Samples were typically prepared in 100 mM sodium phosphate buffer pH 7.4 containing 25% v/v D_2_O and 484 µM TSP in a micro-centrifuge tube. For time-course experiments, cysteamine was added last, and the samples pre-mixed and transferred to 5 mm Norell NMR tubes for analysis. Experiments were performed at 298 K with water suppression unless stated otherwise. Data processing was conducted in Topspin 4.1.4 software, and chemical shifts recorded in ppm relative to the TSP standard (δ_H_ 0.0 ppm).

#### Thiazolidine formation with cysteamine and HCHO (10 eq.)

12 µL of 1 M HCHO in H_2_O (20 mM) was added to 421 µL of 100 mM sodium phosphate buffer pH 7.4, 150 µL of D_2_O and 5 µL of 10 mg/mL TSP in D_2_O in a micro-centrifuge tube. 12 µL of 100 mM cysteamine in H_2_O (2 mM) was added last. The sample was vortex mixed and immediately transferred to an NMR tube for analysis. ^1^H NMR time-course analysis was conducted over 5 h, with 301 s between sample mixing and the first NMR acquisition. The same sample was analysed by ^1^H-^13^C HSQC for 2D characterisation.

#### Thiazolidine formation with cysteamine (2 eq.) and HCHO

6 µL of 0.1 M HCHO in H_2_O (1 mM) was added to 427 µL of 100 mM sodium phosphate buffer pH 7.4, 150 µL of D_2_O and 5 µL of 10 mg/mL TSP in D_2_O in a micro-centrifuge tube. 12 µL of 100 mM cysteamine in H_2_O (2 mM) was added last. The sample was vortex mixed and immediately transferred to an NMR tube for analysis. ^1^H NMR time-course analysis was conducted over 5 h, with 362 s between sample mixing and the first NMR acquisition.

#### Cysteamine analysis

150 µL of D_2_O was added to 433 µL of 100 mM sodium phosphate buffer pH 7.4 and 5 µL of 10 mg/mL TSP in D_2_O in a micro-centrifuge tube. 12 µL of 100 mM cysteamine in H_2_O (2 mM) was added last. The sample was vortex mixed and immediately transferred to an NMR tube for analysis. ^1^H NMR time-course analysis was conducted over 5 h, with 359 s between sample mixing and the first NMR acquisition.

#### Thiazolidine stability (authentic standard)

12 µL of 100 mM thiazolidine in H_2_O (2 mM) was added to 433 µL of 100 mM sodium phosphate buffer pH 7.4, 150 µL of D_2_O and 5 µL of 10 mg/mL TSP in D_2_O in a micro-centrifuge tube. The sample was immediately vortex mixed and transferred to an NMR tube for analysis. ^1^H NMR time-course analysis was conducted over 5 h, with 814 s between sample mixing and the first NMR acquisition. The same sample was analysed by ^1^H-^13^C HSQC for 2D characterisation.

#### Thiazolidine stability after heating (10 eq. HCHO)

Multiple identical samples were prepared as follows: 10 µL of 1 M HCHO in H_2_O (16.7 mM) was added to 425 µL of 100 mM sodium phosphate buffer pH 7.4, 150 µL of D_2_O and 5 µL of 10 mg/mL TSP in D_2_O in a micro-centrifuge tube. 10 µL of 100 mM cysteamine in H_2_O (1.6 mM) was added last and the sample was vortex mixed. Samples were incubated at room temperature for 10 min, and then heated at 80 or 100 °C for 10 min to mimic GC–MS conditions (pre-incubation). Samples were then heated for an additional 10, 20, 30 or 60 min at 80 or 100 °C to mimic GC–MS sample extraction. Samples were then transferred to NMR tubes for analysis. ^1^H NMR time-course analysis was conducted over one hour, with 15–30 min between sample heating and the first NMR acquisition.

#### Thiazolidine stability after heating (2 eq. cysteamine)

Multiple identical samples were prepared as follows: 6 µL of 0.1 M HCHO in H_2_O (1 mM) was added to 427 µL of 100 mM sodium phosphate buffer pH 7.4, 150 µL of D_2_O and 5 µL of 10 mg/mL TSP in D_2_O in a micro-centrifuge tube. 12 µL of 100 mM cysteamine in H_2_O (2 mM) was added last and the sample was vortex mixed. Samples were incubated at room temperature for 10 min, and then heated at 80 or 100 °C for 10 min to mimic GC–MS conditions (pre-incubation). Samples were then heated for an additional 10, 20, 30 or 60 min at 80 or 100 °C to mimic GC–MS sample extraction. Samples were then transferred to NMR tubes for analysis. ^1^H NMR time-course analysis was conducted over one hour, with 12–15 min between sample heating and the first NMR acquisition.

#### Thiazolidine stability after heating (authentic standard)

12 µL of 100 mM thiazolidine in H_2_O (2 mM) was added to 433 µL of 100 mM sodium phosphate buffer pH 7.4, 150 µL of D_2_O and 5 µL of 10 mg/mL TSP in D_2_O in a micro-centrifuge tube. The sample was incubated at room temperature for 10 min, and then heated at 45 °C for 10 min to mimic GC–MS conditions (pre-incubation). The sample was then heated for an additional 60 min at 45 °C to mimic GC–MS sample extraction. The sample was then transferred to an NMR tube for analysis. ^1^H NMR time-course analysis was conducted over 5 h, with 19 min 27 s between sample heating and the first NMR acquisition.

#### Thiazolidine formation at varying concentrations of HCHO

600 µL samples were prepared in micro-centrifuge tubes containing varying concentrations of HCHO (1, 10, 20, 40, 60, 80 100, 500 and 1000 µM) in 100 mM sodium phosphate buffer pH 7.4. 150 µL of D_2_O and 5 µL of 10 mg/mL TSP in D_2_O were then added. 12 µL of 100 mM cysteamine in H_2_O (2 mM) was added last and the samples were vortex mixed. Samples were incubated at room temperature for 30 min, then transferred to an NMR tube for ^1^H NMR analysis.

#### Selectivity analysis (product characterisation)

100 µL of 100 mM carbonyl in 100 mM sodium phosphate buffer pH 7.4 (acetaldehyde, propionaldehyde, butyraldehyde, glyoxylic acid and acetone, 16.7 mM) was added to 335 µL of 100 mM sodium phosphate buffer pH 7.4, 150 µL of D_2_O and 5 µL of 10 mg/mL TSP in D_2_O in a micro-centrifuge tube. 10 µL of 100 mM cysteamine in H_2_O (2 mM) was added last, and the samples were vortex mixed and immediately transferred to an NMR tube for analysis. ^1^H NMR time-course analysis was conducted over one hour, with 190–360 s between sample mixing and the first NMR acquisition. Samples were re-analysed by ^1^H NMR after 24 h.

#### Selectivity analysis (competition experiment 1)

Stock: 200 µL of 1 M carbonyl in 100 mM sodium phosphate buffer pH 7.4 (acetaldehyde, propionaldehyde or butyraldehyde, 100 equivalents) was added to 650 µL of 100 mM sodium phosphate buffer pH 7.4, 300 µL of D_2_O and 10 µL of 10 mg/mL TSP in D_2_O in a micro-centrifuge tube. 20 µL of 100 mM cysteamine in H_2_O (1 equivalent) was added last, and the sample was vortex mixed. A: 10 µL of H_2_O was added to 590 µL of the stock and the sample was vortex mixed and immediately transferred to an NMR tube for analysis. ^1^H NMR time-course analysis was conducted over one hour, with 200–300 s between sample mixing and the first NMR acquisition. B: 10 µL of 1 M HCHO in H_2_O (10 equivalents) was added to 590 µL of the stock and the sample was vortex mixed and immediately transferred to an NMR tube for analysis. ^1^H NMR time-course analysis was conducted over one hour, with 200–300 s between sample mixing and the first NMR acquisition.

#### Selectivity analysis (competition experiment 2)

Stock: 20 µL of 1 M HCHO in 100 mM sodium phosphate buffer pH 7.4 (10 equivalents) was added to 650 µL of 100 mM sodium phosphate buffer pH 7.4, 300 µL of D_2_O and 10 µL of 10 mg/mL TSP in D_2_O in a micro-centrifuge tube. 20 µL of 100 mM cysteamine in H_2_O (1 eq.) was added last, and the sample was vortex mixed. A: 100 µL of 100 mM sodium phosphate buffer pH 7.4 was added to 500 µL of the stock and the sample was vortex mixed and immediately transferred to an NMR tube for analysis. ^1^H NMR time-course analysis was conducted over one hour, with 200–265 s between sample mixing and the first NMR acquisition. B: 100 µL of 1 M carbonyl in 100 mM sodium phosphate buffer pH 7.4 (acetaldehyde, propionaldehyde or butyraldehyde, 100 eq.) was added to 500 µL of the stock and the sample was vortex mixed and immediately transferred to an NMR tube for analysis. ^1^H NMR time-course analysis was conducted over one hour, with 200–300 s between sample mixing and the first NMR acquisition.

#### Product formation with PFBHA and HCHO (10 eq.)

12 µL of 1 M HCHO in H_2_O (20 mM) was added to 421 µL of 100 mM sodium phosphate buffer pH 7.4, 150 µL of D_2_O and 5 µL of 10 mg/mL TSP in D_2_O in a micro-centrifuge tube. 12 µL of 100 mM PFBHA in H_2_O (2 mM) was added last. The sample was vortex mixed and immediately transferred to an NMR tube for analysis. ^1^H NMR time-course analysis was conducted over 5 h, with 752 s between sample mixing and the first NMR acquisition. The same sample was re-analysed every 24 h for 7 days.

#### Product formation with PFBHA (2 eq.) and HCHO

6 µL of 0.1 M HCHO in H_2_O (1 mM) was added to 427 µL of 100 mM sodium phosphate buffer pH 7.4, 150 µL of D_2_O and 5 µL of 10 mg/mL TSP in D_2_O in a micro-centrifuge tube. 12 µL of 100 mM PFBHA in H_2_O (2 mM) was added last. The sample was vortex mixed and immediately transferred to an NMR tube for analysis. ^1^H NMR time-course analysis was conducted over 5 h, with 980 s between sample mixing and the first NMR acquisition. The same sample was re-analysed every 24 h for 7 days.

#### PFBHA competition experiment 1

Stock: 20 µL of 10 mM HCHO in water, (1 equivalent) was added to 650 µL of 100 mM sodium phosphate buffer pH 7.4, 300 µL of D_2_O and 10 µL of 10 mg/mL TSP in D_2_O in a micro-centrifuge tube. 20 µL of 50 mM cysteamine in H_2_O:DMSO-d_6_ (5 equivalents) was added last, and the sample was vortex mixed. A: 100 µL of 100 mM sodium phosphate buffer pH 7.4 was added to 500 µL of the stock and the sample was vortex mixed and immediately transferred to an NMR tube for analysis. ^1^H NMR time-course analysis was conducted over one hour, with 377 s between sample mixing and the first NMR acquisition. B: 100 µL of 50 mM PFBHA in H_2_O:DMSO-d_6_ (50 eq.) was added to 500 µL of the stock and the sample was vortex mixed and immediately transferred to an NMR tube for analysis. ^1^H NMR time-course analysis was conducted over one hour, with 406 s between sample mixing and the first NMR acquisition.

#### PFBHA competition experiment 2

Stock: 20 µL of 10 mM HCHO in water, (1 equivalent) was added to 650 µL of 100 mM sodium phosphate buffer pH 7.4, 300 µL of D_2_O and 10 µL of 10 mg/mL TSP in D_2_O in a micro-centrifuge tube. 20 µL of 50 mM PFBHA in H_2_O:DMSO-d_6_ (5 equivalent) was added last, and the sample was vortex mixed. A: 100 µL of 100 mM sodium phosphate buffer pH 7.4 was added to 500 µL of the stock and the sample was vortex mixed and immediately transferred to an NMR tube for analysis. ^1^H NMR time-course analysis was conducted over 1 h, with 415 s between sample mixing and the first NMR acquisition. B: 100 µL of 50 mM cysteamine in H_2_O:DMSO-d_6_ (50 equivalents) was added to 500 µL of the stock and the sample was vortex mixed and immediately transferred to an NMR tube for analysis. ^1^H NMR time-course analysis was conducted over one hour, with 402 s between sample mixing and the first NMR acquisition.

### SPME GC–MS experiments

#### General

SPME GC–MS experiments were conducted on a 7890A GC and 5975C MS (Agilent) fitted with a CTC-PAL auto-sampler. Validation and cell experiments were conducted on a 8890 GC (Agilent) and Pegasus BT 4D GC x GC TOF MS (LECO) fitted with a CTC-PAL auto-sampler. Samples were treated with a pre-incubation for 10 min at 500 rpm at either 100 °C (headspace) or 45 °C (immersive) prior to extraction with the Arrow fibre (Carbon WR/PDMS, 1.1 mm × 120 µm, Agilent). The fibre was pre-conditioned (300 °C, 5 min) before immersion in the GC sample vial (2 mL, Agilent). Sample extraction was conducted at 100 °C with 11 mm vial penetration (headspace) or 45 °C with 22 mm vial penetration (immersive) for 60 min. Desorption into the GC inlet was then conducted (300 °C, 3 min) before column separation (DB-WAX capillary column, 30 m × 250 µm × 0.25 µm, Agilent). The fibre was agitated in water for 5 min between samples for immersive extraction.

GC conditions: initial column temperature was 40 °C (5 min), raised to 250 °C at 7 °C/min. The total GC run time was 40 min, with the GC inlet operated in split (7:1) or splitless mode at 250 °C. Helium was used as the carrier gas (1 mL/min flow rate, septum purge flow rate of 3 mL/min (split) or 2 mL/min (splitless)).

MS: the instrument was set to SIM mode for the thiazolidine ions *m/z* 89, *m/z* 59 and *m/z* 43 (*m/z* 89 was used for quantification), as well as *m/z* 90 for the internal standard (retention time: 16.8 min). A dwell time of 100 ms was used for each ion. The MS source temperature was set to 230 °C, and the quadrupole temperature was set to 150 °C.

GC–MS data was acquired using MassHunter GC–MS Acquisition software, with MassHunter Quantitative Analysis software used for data processing. Data analysis was conducted in Microsoft Excel and GraphPad Prism. Samples were prepared in 100 mM sodium phosphate buffer pH 7.4 in 2 mL glass GC vials (Agilent) at a final volume of 1 mL. Samples were stored at room temperature and analysed within 7 days of preparation.

#### Headspace SPME extraction

Calibration standards were prepared containing varying concentrations of HCHO across a concentration range of 10 nM–2 mM in 100 mM sodium phosphate buffer pH 7.4. 20 µL of 100 mM cysteamine (2 mM) was added last and the samples vortex mixed prior to analysis. The final sample volume was 0.5 mL. Samples were analysed by headspace SPME GC–MS, with identical calibration samples heated at 80 or 100 °C for either 10, 20, 30 or 60 min for sample extraction (all samples were heated for an additional 10 min (pre-incubation) prior to fibre immersion in the headspace to equilibrate the samples to the extraction temperature).

#### Immersive SPME extraction

Calibration standards were prepared containing varying concentrations of HCHO across a concentration range of 1 nM–1 mM in 100 mM sodium phosphate buffer pH 7.4. 20 µL of 100 mM cysteamine (2 mM) was added last and the samples vortex mixed prior to GC–MS analysis. The final sample volume was 0.5 mL. Samples were analysed by immersive SPME GC–MS, with sample extraction for 60 min at 45 °C (samples were heated for an additional 10 min (pre-incubation) prior to fibre immersion in the sample to equilibrate the samples to the extraction temperature).

#### Method validation

All samples for method validation were analysed using the optimised immersive SPME method (45 °C, 60 min sample extraction). Calibration standards were prepared across a concentration range of 10 µM–1 mM (8 standards) in 100 mM sodium phosphate buffer pH 7.4. 100 mM cysteamine was added to each sample to a final concentration of 2 mM, with 500 µM of internal standard (IS, ^13^C-labelled thiazolidine) added last. Samples were vortex mixed prior to analysis, and all calibration standards analysed in duplicate (at the start and end of each batch). An additional standard 1 (10 µM) was prepared as a system suitability test (SST). The R^2^ of the calibration curve was determined to be 0.998. Quality control (QC) samples were prepared in a similar manner, with different HCHO stocks than those used to prepare the calibration standards. QCs were prepared at four HCHO concentrations—50 µM (Low), 100 µM (Medium Low), 500 µM (Medium High) and 1 mM (High). 2 mM cysteamine and 500 µM IS were also added. QC samples were analysed in 6 replicates and used for an intra-batch precision and accuracy assessment. The coefficient of variation (%CV) and relative error (%RE) were calculated to be 19.4 and − 30.4 at QC Low, 7.39 and − 8.79 at QC Medium Low, 0.829 and − 2.63 at QC Medium High and 1.25 and − 9.18 at QC High respectively, with the majority falling within the validation acceptance criteria of ± 15% (± 20% at QC Low). Blank buffer samples were analysed after calibration curves and QC High samples to minimise carryover.

#### Bacterial cell analysis

25 µL of BL21 (DE3) cells were added to 200 µL 2XYT media in a sterile micro-centrifuge tube. The sample was incubated at 37 °C, 180 rpm for 1 h. 50 µL was spread onto an LB/agar plate, and the plate incubated at 37 °C overnight. A single colony was inoculated in 100 mL of LB media and incubated at 37 °C, 180 rpm overnight (OD_600_ 3.75 of the overnight culture). The culture was centrifuged (20 min, 5000 rpm) to harvest a single pellet, and the supernatant discarded. The pellet was re-suspended in 25 mL of 100 mM sodium phosphate buffer pH 7.4 containing a single protease inhibitor tablet. The cells were lysed by sonicated (30 s on, 30 s off, 6 cycles, 12 microns) before treatment with DNase 1 and lysozyme (stirred for 20 min at 4 °C). The lysate was centrifuged (50 min, 18,000 rpm), and the supernatant sterile filtered (0.2 µm). Protein was precipitated from the lysate using 75 mL of acetonitrile (3:1 acetonitrile:buffer), which produced a white precipitate. The lysate was left stirring for 30 min at 4 °C before being centrifuged (20 min, 18,000 rpm). The supernatant was used for GC–MS sample preparation.

Calibration standards and QC samples were prepared in buffer for the batch as described previously. For bacterial samples, preparation was identical to calibration standards, with cell lysate replacing the buffer. HCHO was added to the lysate to prepare a pseudo-calibration curve. Additionally, each cell calibration standard was further diluted (1:2) in buffer 5 times to assess the effects of dilution on HCHO quantification. Cysteamine and IS were added to final concentrations of 2 mM and 500 µM respectively, and samples analysed in triplicate by immersive SPME GC–MS as described previously. Additional acetonitrile QC samples were also prepared and analysed in duplicate, with samples prepared at four concentrations as described above, with 3:1 acetonitrile:buffer replacing 100% buffer.

### Equations

#### Precision (%CV)

$$\%CV = \left(\frac{S.D.}{Mean}\right) \times 100.$$

#### Accuracy (%RE)

$$\%RE =\left(\frac{Measured \,conc. - Calculated\, conc.}{Calculated\, conc.}\right) \times 100.$$

### Supplementary Information

Supplementary Figures.

## Data Availability

All data pertinent to this study are available in the main article and in the Supplementary Information. All data are available from the corresponding author on reasonable request.
