# Supplementary material for: Quantitative detection of formaldehyde using solid phase microextraction gas chromatography–mass spectrometry coupled to cysteamine scavenging
Source: Sci Rep. 2023 Sep 5;13:14642. doi: 10.1038/s41598-023-41609-0 (PMC10480157; doi:10.1038/s41598-023-41609-0)
Supplement: Supplementary file 1 — Supplementary Figures. [file 41598_2023_41609_MOESM1_ESM.pdf]

Supplementary Information

Quantitative Detection of Formaldehyde using Solid Phase Microextraction Gas Chromatography-Mass Spectrometry coupled to Cysteamine Scavenging

Sara Y. Chothia,[1] Matthew Carr,[1] Paul S. Monks,[2] Rebecca L. Cordell[2] and Richard J. Hopkinson*[1]

*[1]Leicester Institute for Structural and Chemical Biology and School of Chemistry, University of Leicester, Henry Wellcome Building, Lancaster Road, Leicester, LE1 7RH, UK. *E-mail: richard.hopkinson@leicester.ac.uk*

[2]Space Park Leicester, University of Leicester, 92 Corporation Road, Leicester, LE4 5SP, UK

Supplementary Figures

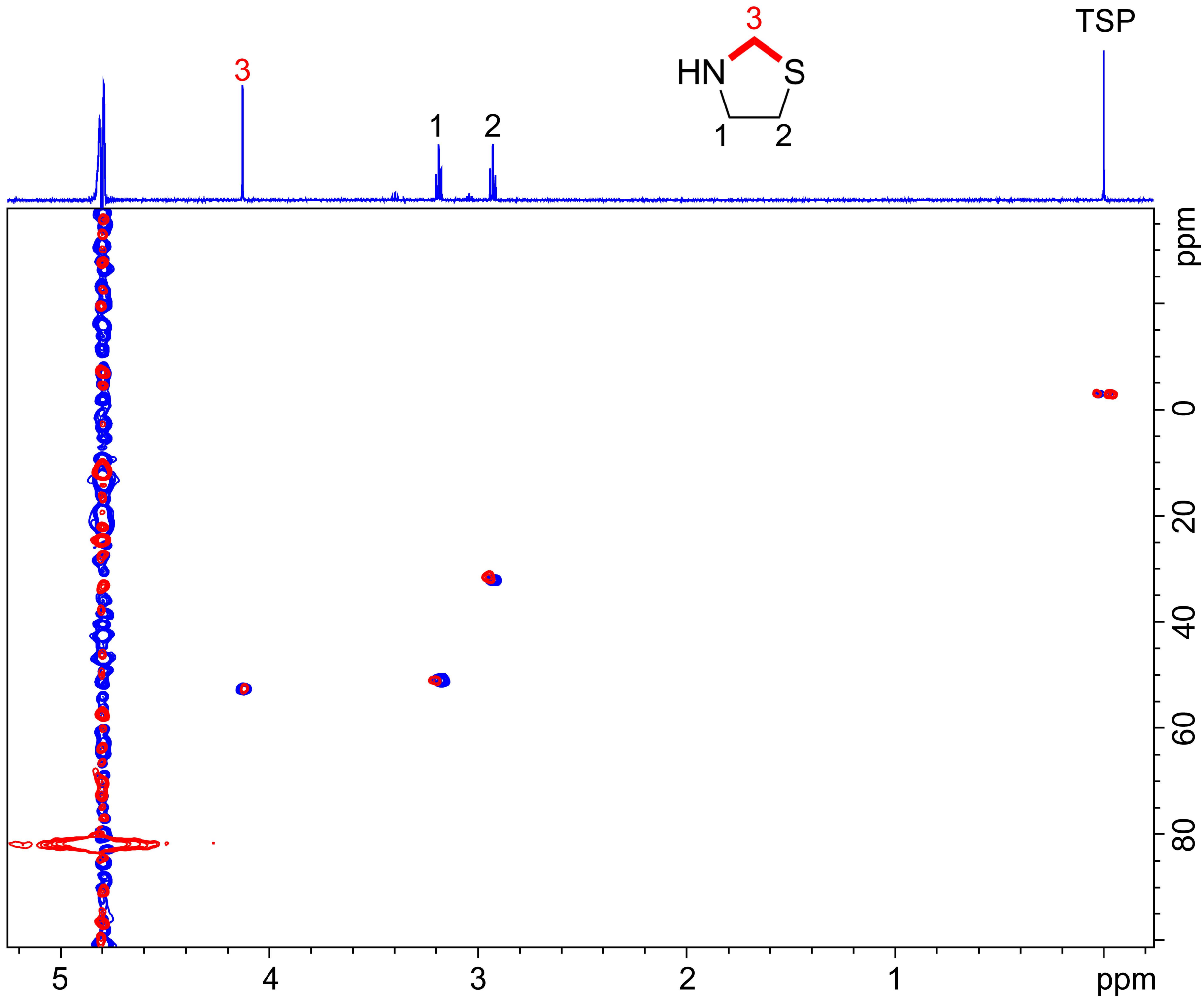

Figure S1. ^{1}H-^{13}C-HSQC spectrum of cysteamine incubated with a 10-fold of HCHO in 100 mM sodium phosphate buffer pH 7.4 (containing 25 % v/v D_2O). Resonances corresponding to HCHO-derived thiazolidine are highlighted. A ^{1}H-^{13}C-HSQC spectrum of authentic HCHO-derived thiazolidine is overlaid (blue).

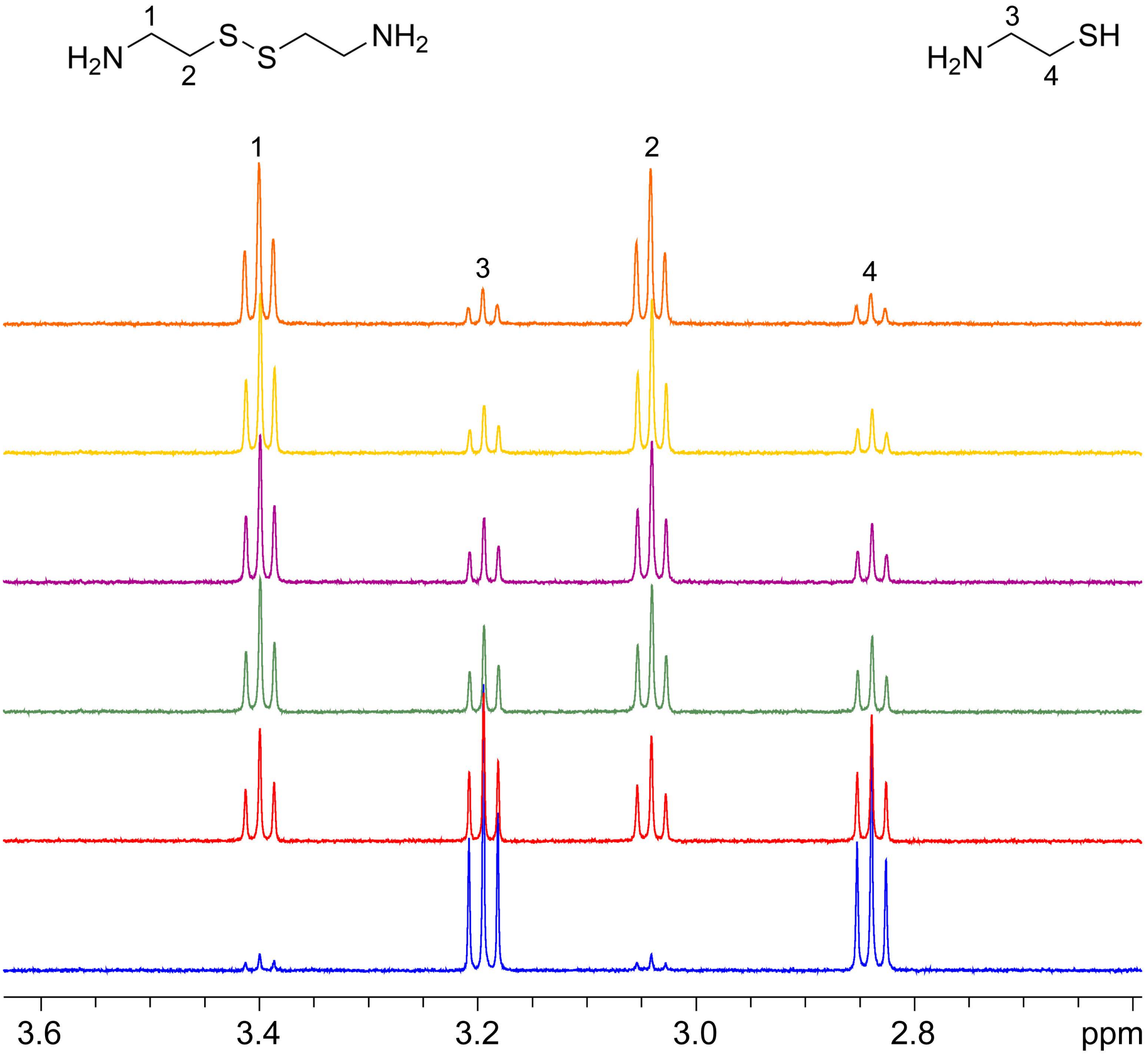

Figure S2. ^{1}H NMR spectra of a sample of cysteamine in 100 mM sodium phosphate buffer pH 7.4 (containing 25 % v/v D_2O) over time at 25 °C. Time-dependent formation of cystamine is observed. Resonances corresponding to cysteamine and cystamine are highlighted.

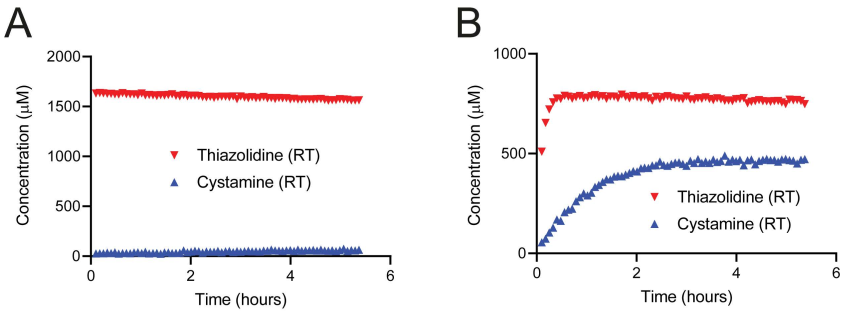

Figure S3. Graphs showing time-dependent degradation of HCHO-derived thiazolidine and formation of cystamine over time. (A) Time-course analysis of a sample initially containing cysteamine (2 mM) and HCHO (20 mM). Only low-level thiazolidine degradation and cystamine formation is observed (B) Time-course analysis of a sample initially containing cysteamine (2 mM) and HCHO (1 mM). Cystamine formation is significantly increased; however, the rate of thiazolidine degradation is comparable to that observed with excess HCHO.

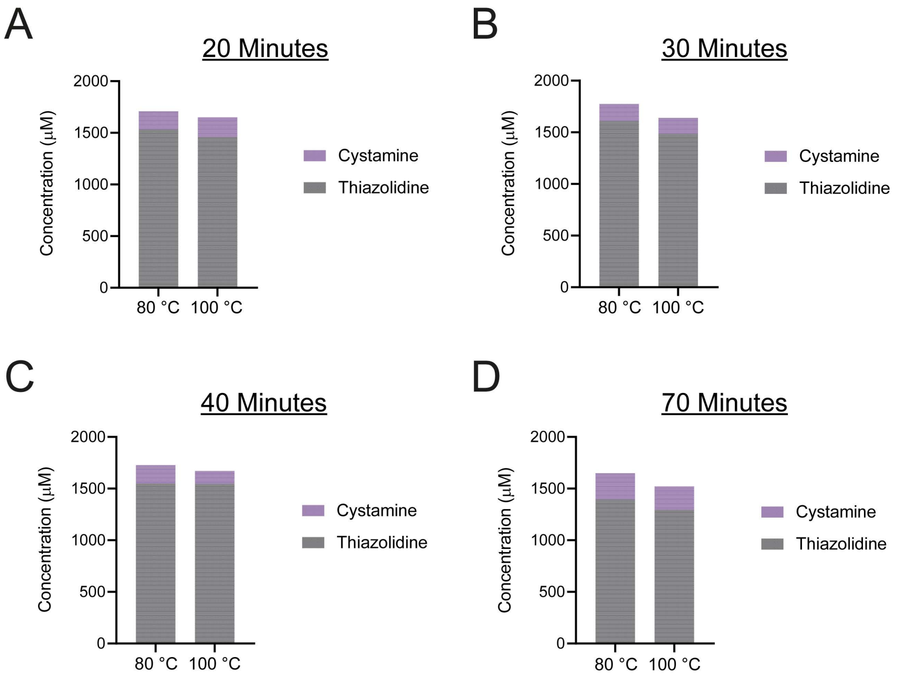

Figure S4. Bars graphs showing concentrations of HCHO-derived thiazolidine and cystamine in samples initially containing cysteamine (1.67 mM) and HCHO (16.7 mM), heated at either 80 °C or 100 °C for 20, 30, 40 and 70 minutes respectively. Most significant thiazolidine degradation was observed after long heating times.

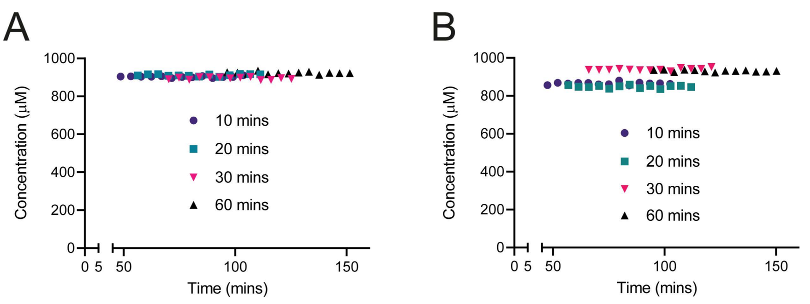

Figure S5. Graphs showing concentrations of HCHO-derived thiazolidine in samples initially containing cysteamine (2 mM) and HCHO (1 mM), heated at either 80 °C or 100 °C for 20, 30, 40 and 70 minutes respectively. Only low-level degradation is observed.

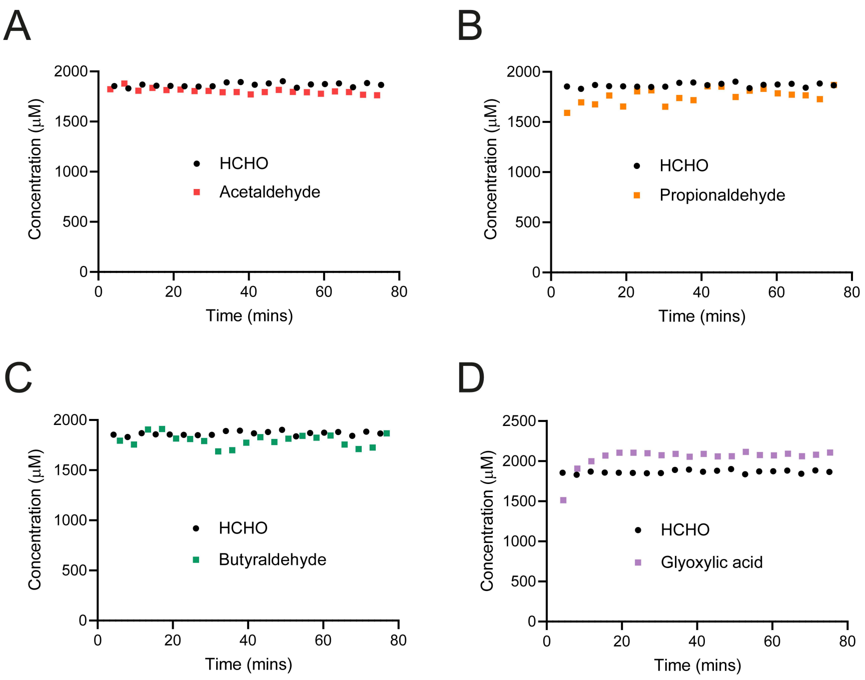

Figure S6. Time-course graphs showing concentrations of thiazolidines formed in samples containing cysteamine and 10-fold excesses of either acetaldehyde (A), propionaldehyde (B), butyraldehyde (C) and glyoxylic acid (D).

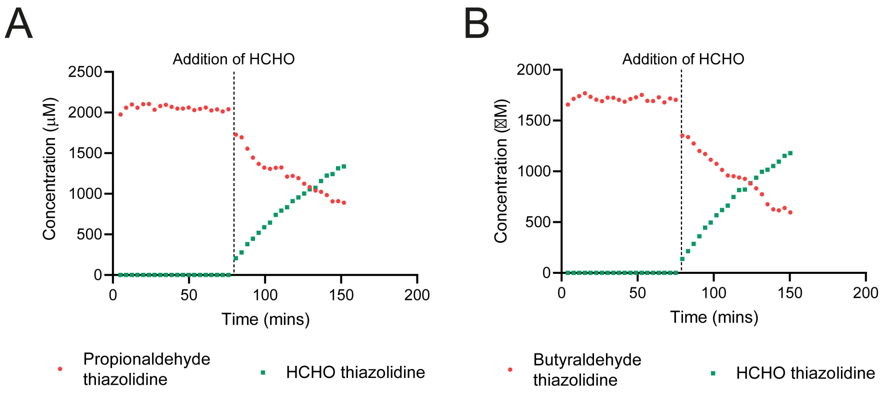

Figure S7. Graphs showing thiazolidine concentrations in samples of cysteamine first incubated (A) with propionaldehyde (10-fold excess) followed by addition of HCHO (50-fold excess), or (B) with butyraldehyde (10-fold excess) followed by addition of HCHO (50-fold excess). In the absence of HCHO, full conversion to the propionaldehyde- or butyraldehyde-derived thiazolidine is observed; however, addition of HCHO leads to rapid conversion to the HCHO-derived thiazolidine.

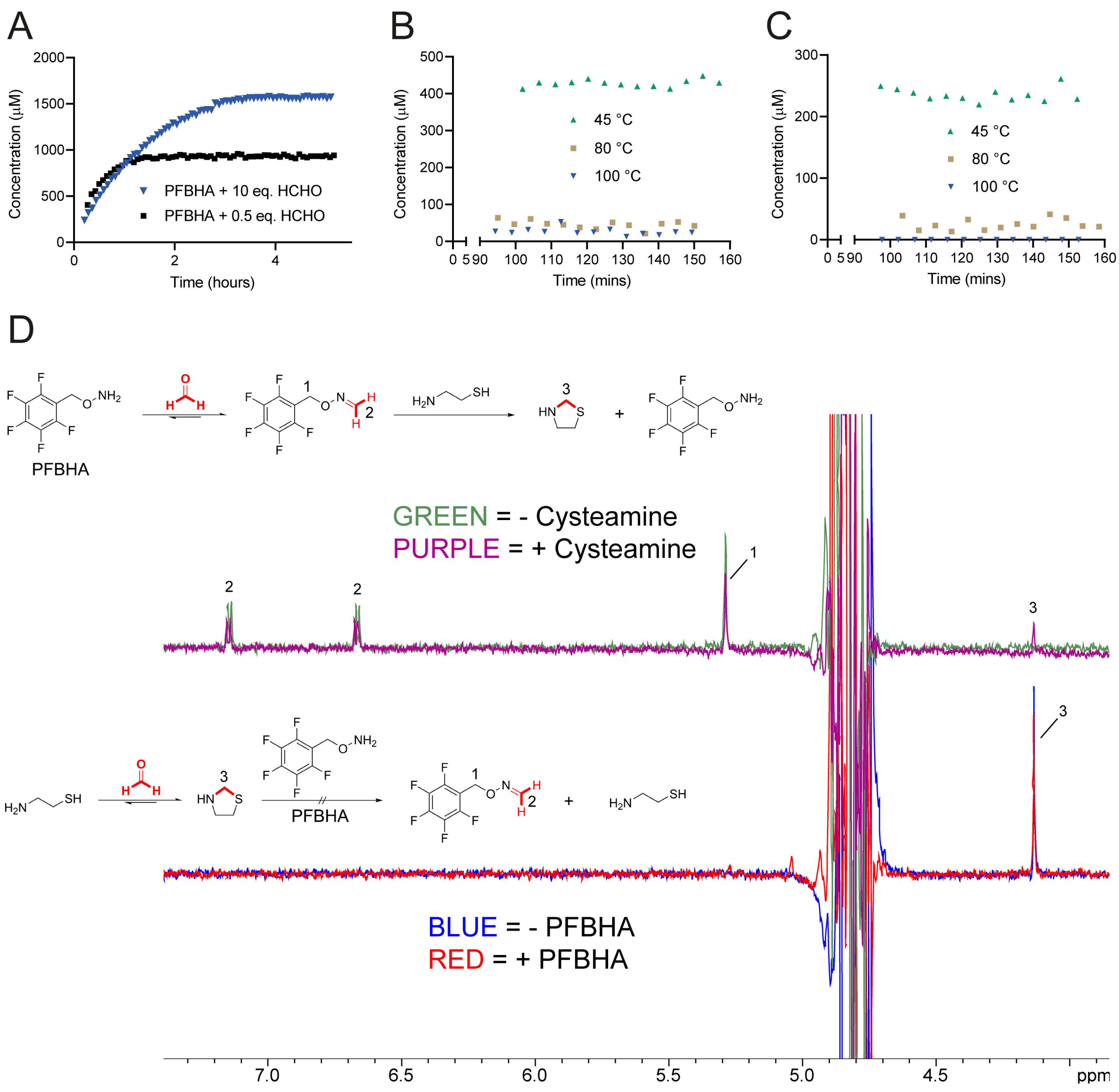

Figure S8. (A) Graphs showing time-dependent formation of the PFBHA-HCHO adduct (an oxime) in samples containing PFBHA (2 mM) and HCHO at either 1 mM (black) or 20 mM (blue) (B) Graph showing concentrations of HCHO-derived thiazolidine in samples initially containing cysteamine (2 mM) and HCHO (20 mM), heated at either 45 °C, 80 °C or 100 °C for 70 minutes. (C) Graph showing concentrations of HCHO-derived thiazolidine in samples initially containing cysteamine (2 mM) and HCHO (1 mM), heated at either 45 °C, 80 °C or 100 °C for 70 minutes. (D) ^{1}H NMR spectra showing competition experiments between cysteamine and PFBHA for reaction with HCHO. When cysteamine was reacted first with HCHO, followed by addition of PFBHA, no formation of the PFBHA-derived oxime was observed (blue and

red). Integration of the ^{1}H resonance at δ_H 4.1 ppm (corresponding to the thiazolidine) revealed no loss of intensity after addition of PFBHA. However, both the oxime and the cysteamine-derived thiazolidine were observed when PFBHA was reacted first (green and purple), while resonances corresponding to the oxime (at δ_H 5.3 ppm, δ_H 6.6 ppm and δ_H 7.1 ppm) decreased in intensity after addition of cysteamine.

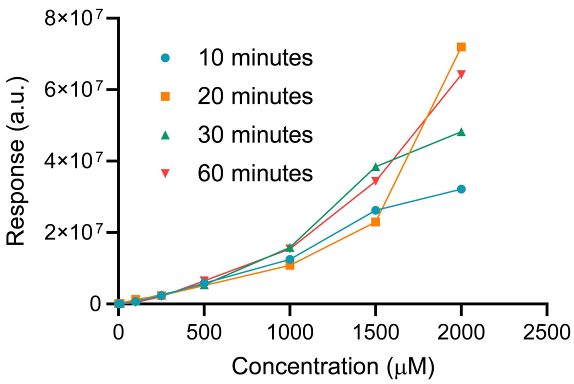

Figure S9. Graph showing headspace GC-MS response curves from samples of cysteamine incubated with HCHO. The samples were heated at 80 °C for either 10, 20, 30 or 60 minutes to induce evaporation of thiazolidine into the headspace and adsorption onto the SPME fibre (note: the samples were also subjected to a pre-incubation step involving heating at 80 °C for 10 minutes). a.u = arbitrary units.

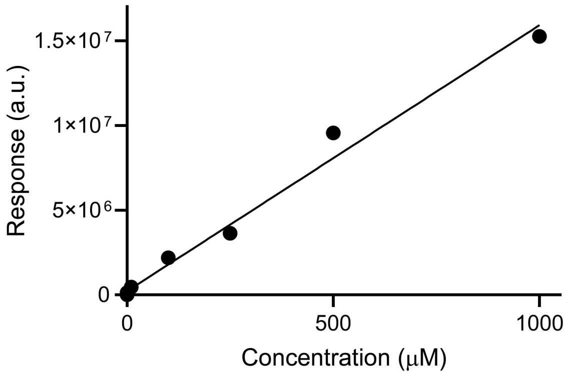

Figure S10. Calibration curve of HCHO-derived thiazolidine using headspace SPME extraction (100 °C, 70 minutes) and GC-MS analysis. R^2 = 0.987.

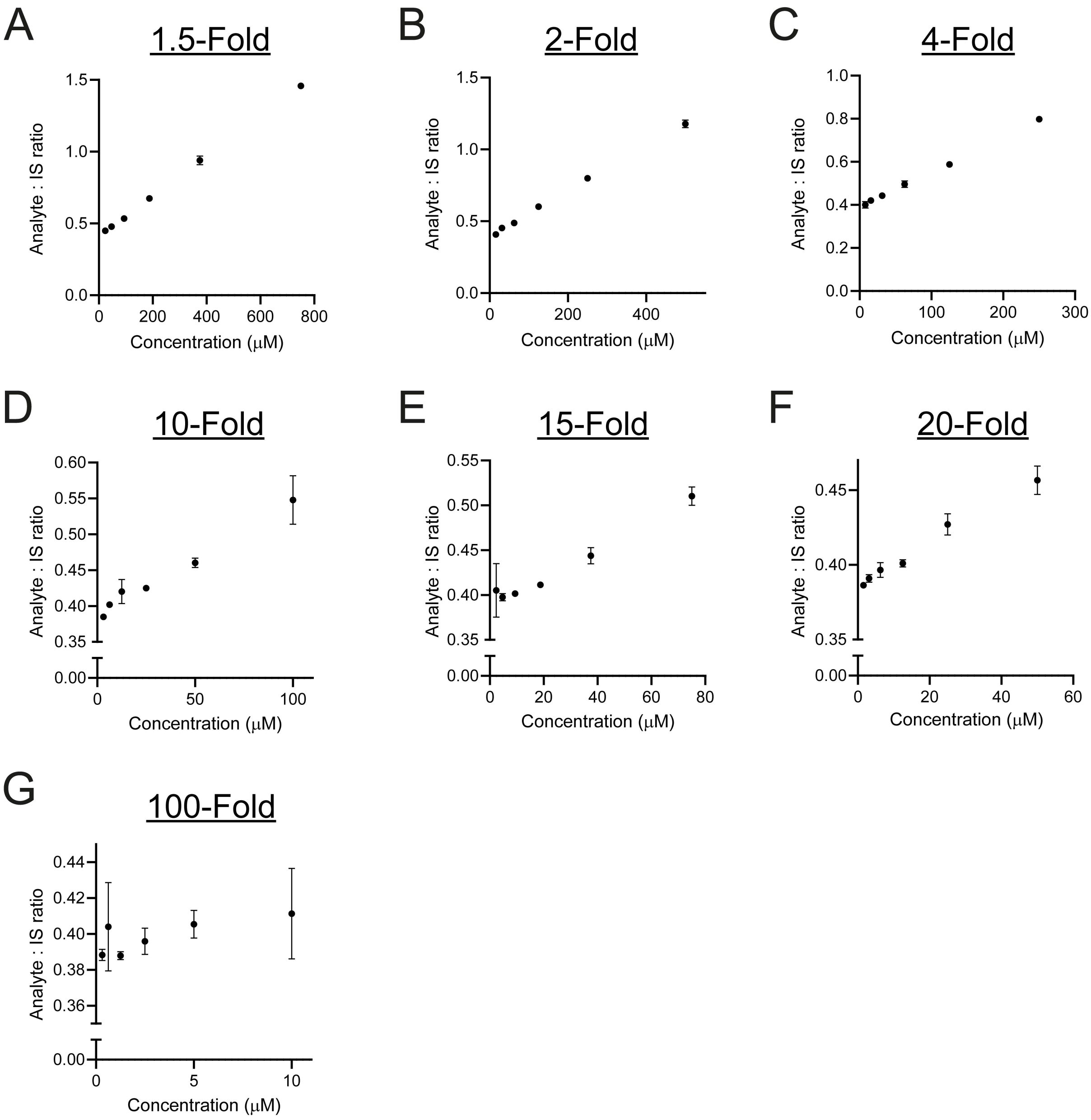

Figure S11. Calibration curves of HCHO-derived thiazolidine in *E. coli* cell lysate extracted using immersive SPME extraction (45 °C, 70 minutes). Each sample was diluted with 100 mM sodium phosphate buffer pH 7.4 prior to extraction: (A) 1.5-fold, (B) 2-fold, (C) 4-fold, (D) 10-fold, (E) 15-fold, (F) 20-fold, (G) 100-fold. Error bars represent standard deviations (n = 3).
